# Ongoing transmission of trachoma in low prevalence districts in Mozambique: results from four cross-sectional enhanced impact surveys, 2022

**DOI:** 10.1038/s41598-024-71201-z

**Published:** 2024-10-15

**Authors:** Henis Mior Sitoe, William E. Oswald, Felizmina Zita, Mawo Fall, Tamimo Momade, Molly W. Adams, Rebecca M. Flueckiger, Scott McPherson, Sabrina Eyob, Thuy Doan, Thomas M. Lietman, Benjamin F. Arnold, Karana Wickens, Sarah Gwyn, Diana L. Martin, Mabula Kasubi, Sarah Boyd, Ana Bakhtiari, Cristina Jimenez, Anthony W. Solomon, Emma M. Harding-Esch, Upendo J. Mwingira, Jeremiah M. Ngondi

**Affiliations:** 1 Ministério da Saúde, Maputo, Mozambique; 2 RTI International, Research Triangle Park, USA; 3 RTI International, Maputo, Mozambique; 4 F.I. Proctor Foundation and University of California San Francisco, San Francisco, USA; 5 Oak Ridge Institute for Science and Education, Oak Ridge, USA; 6 Centers for Disease Control and Prevention, Atlanta, USA; 7 Muhimbili University of Health and Allied Sciences, Dar es Salaam, Tanzania; 8 International Trachoma Initiative, Task Force for Global Health, Decatur, USA; 9 Sightsavers, Haywards Heath, UK; 10 Global Neglected Tropical Diseases Programme, World Health Organization, Geneva, Switzerland; 11 London School of Hygiene & Tropical Medicine, London, UK

**Keywords:** Bacterial infection, Diagnostic markers, Epidemiology, Population screening, Conjunctival diseases

## Abstract

Mozambique is making progress towards elimination of trachoma as a public health problem, but in some districts trachomatous inflammation—follicular (TF) prevalence remains above the 5% elimination threshold despite years of various interventions, including antibiotic mass drug administration. To characterize transmission in four districts, we incorporated testing of ocular infection and serology into routine trachoma impact surveys (TIS) in August 2022. We examined residents aged ≥ 1 year for trachoma and collected information on household water, sanitation, and hygiene. Among children aged 1–9 years, we tested conjunctival swabs for *Chlamydia trachomatis* nucleic acid and dried blood spots for *C. trachomatis* antibodies. We modeled age-dependent seroprevalence to estimate seroconversion rate (SCR). We examined 4841 children aged 1–9 years. TF prevalence ranged between 1.1 and 6.0% with three districts below the 5% threshold. PCR-confirmed infection prevalence ranged between 1.1 and 4.8%, and Pgp3 seroprevalence ranged between 8.8 and 24.3%. Pgp3 SCR was 1.9 per 100 children per year in the district with the lowest TF prevalence. Two other districts with TF < 5% had SCR of 5.0 and 4.7. The district with TF ≥ 5% had a SCR of 6.0. This enhanced TIS furthered understanding of transmission in these districts and provides information on additional indicators for monitoring trachoma programs.

## Introduction

Trachoma remains the leading infectious cause of blindness globally^1^. This neglected tropical disease is caused by repeated ocular infections with the bacterium *Chlamydia trachomatis* (Ct) during childhood, which can result in scarring of the conjunctivae, leading to trichiasis (in-turning of eyelashes that scratch the eyeball) and potentially blindness through resulting damage to the cornea^2^. Global efforts to eliminate trachoma as a public health problem by 2030^3^ rely on interventions in four areas, comprising the SAFE strategy: (S)urgery for the correction of trichiasis; (A)ntibiotics for treatment of the infection, usually through annual mass drug administration (MDA) of azithromycin; (F)acial cleanliness to reduce spread of Ct in ocular or nasal discharge; and (E)nvironmental improvement, including fly control via sanitation and increased access to water for hygiene^4–6^.

Through implementation of the SAFE strategy, as of July 2023, trachoma has been eliminated as a public health problem in 18 formerly endemic countries^7,8^. Elimination of trachoma as a public health problem is defined as (1) a prevalence of trachomatous trichiasis (TT) unknown to the health system of < 0.2% among people aged ≥ 15 years, (2) a prevalence of trachomatous inflammation—follicular (TF) of < 5% among children aged 1–9 years in each formerly endemic district, and (3) evidence that the health system can continue to identify and manage incident cases of TT^9^. Evidence of elimination at the country level is based on clinical examination during population-based prevalence surveys for the presence of TF and TT, using the World Health Organization (WHO) simplified grading system^10,11^. Following a period of implementation of the A, F, and E components of SAFE, trachoma impact surveys (TIS) are conducted at least 6 months after the final MDA round, and then pre-validation trachoma surveillance surveys (TSS) are conducted at least two years after the TIS has reported a TF prevalence < 5% in children aged 1–9 years to demonstrate that trachoma has not re-emerged^9^.

Trachoma programs are confronting a growing proportion of districts which, following implementation of the A, F, and E components of SAFE, have not progressed to elimination^12^. In these settings, TF may be considered persistent in districts in which prevalence has not been below 5% after two or more TIS. Alternatively, TF may be considered recrudescent in districts in which prevalence has returned above 5% during the surveillance period following the last MDA.^12^ Potential factors contributing to persistent or recrudescent TF in these districts are probably varied, and could include: (1) insufficient program delivery of A, F, or E; (2) ineffective MDA due to high baseline TF or macrolide resistance in Ct; and (3) concerns about TF accurately reflecting the actual Ct burden, either due to the imperfect specificity of TF or imprecision of measurement around the 5% TF threshold^12^.

Though TF prevalence has been a useful metric for trachoma elimination efforts to date, there are known limitations to its use as a marker of ocular Ct infection, and new challenges have appeared as implementation of the SAFE strategy has continued. TF does not consistently correlate well with ocular Ct infection, particularly following MDA, and falling TF prevalence globally makes training surveyors in the use of the WHO simplified grading system more difficult^2^. As a result, accurately estimating trachoma prevalence for decision-making in settings nearing elimination targets may require the use of new diagnostic approaches. Available nucleic acid amplification tests for Ct and detection of anti-Ct antibodies through serological testing are promising additional approaches for programmatic decision-making and surveillance at the population level^13–19^. A need exists for more data to understand the relationship between seroprevalence, seroconversion, ocular Ct infection, and TF prevalence in settings at different stages of the elimination process and determine thresholds for decision-making based on these measures^20,21^.

Mozambique conducted trachoma baseline surveys between 2011 and 2015^22^. Based on these, the SAFE strategy was implemented towards the goal of eliminating trachoma as a public health problem, allowing 54 of the 66 endemic districts to discontinue MDA campaigns by 2022, having attained TF prevalence < 5%; however, persistent and recrudescent TF represents a new challenge for the country. As of January 2022, there were six districts with persistent TF and six districts with recrudescent TF. In these districts, it is unclear whether the classification based on TF prevalence was overestimated due to sampling error or whether the observed follicles leading to diagnoses of TF were caused by another pathogen or ocular Ct infection. This study incorporated testing of ocular Ct infection and serology into a routine TIS in four districts in August 2022. We aimed to determine if TF indicates ocular Ct infection in districts anticipated to have low TF prevalence in Mozambique and compare population-level measures of trachoma transmission based on antibody response with measures of infection prevalence**.**

## Methods

### Ethics and consent

This study was conducted in accordance with the Declaration of Helsinki. Approval for the enhanced TIS activity was provided by the Mozambique National Bioethics Committee for Health (462/CNBS/22) and the University of California San Francisco Institutional Review Board (21-35587). Informed consent was obtained from parents/carers for all children, and children aged 6–9 years were asked to provide assent. In addition to household level consent forms signed by parents/carers, consent was also documented electronically on the Tropical Data App for every child. The London School of Hygiene & Tropical Medicine provided ethical approval (16105) for Tropical Data support for the routine TIS. Study staff at the US Centers for Disease Control and Prevention (CDC) did not have access to identifying information and were not considered to be engaged in human subjects research.

### Study sites and survey sampling procedure

**Fig. 1:**
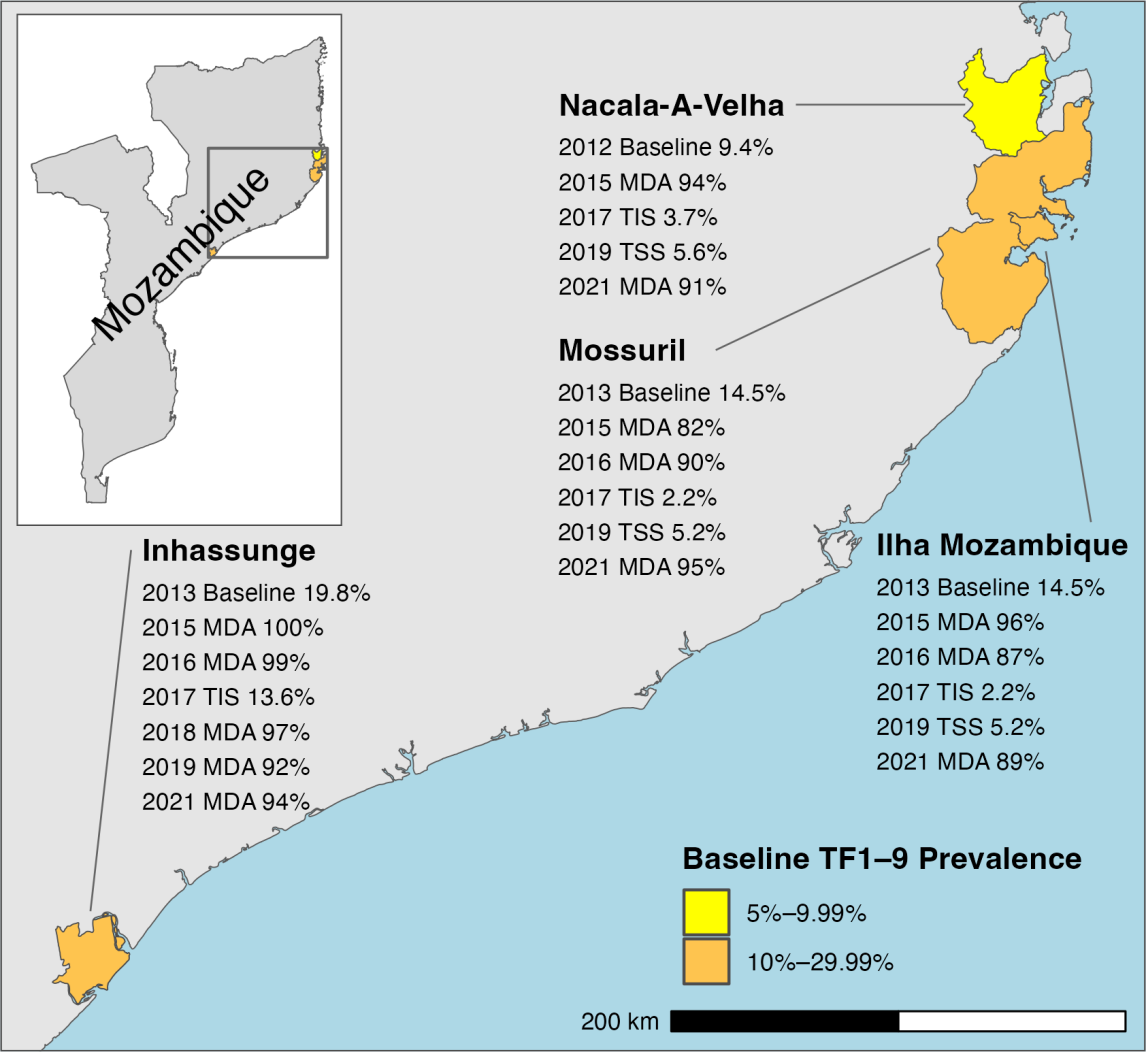
Mass drug administration (MDA) years and reported coverage and historical prevalence of trachomatous inflammation—follicular in children aged 1–9 years (TF1–9) of districts included in an enhanced Trachoma Impact Survey (TIS) in Mozambique in 2022 (Shapefile data available from https://data.humdata.org/dataset/cod-ab-moz, accessed January 26, 2024). TSS = Trachoma Surveillance Survey. The boundaries and names shown and the designations used on this map do not imply the expression of any opinion whatsoever on the part of the authors, or the institutions with which they are affiliated, concerning the legal status of any country, territory, city or area or of its authorities, or concerning the delimitation of its frontiers or boundaries.

WHO recommends that population based-prevalence surveys be conducted at the district or evaluation unit level, which for trachoma elimination purposes is defined as “the normal administrative unit for health care management, consisting of a population unit between 100,000–250,000 persons”^23,24^. We conducted the current enhanced TIS in four evaluation units: Ilha Mozambique; Mossuril; Nacala-A-Velha; and Inhassunge (Fig. 1). Baseline TF prevalences in these districts were 9.4% in Nacala-A-Velha, 14.5% in Ilha Mozambique and Mossuril (formerly in the same evaluation unit), and 19.8% in Inhassunge. Implementation of the A, F, and E components of SAFE had been initiated as baseline TF prevalence was ≥ 5% in all districts. Reported coverage was > 80% for all MDA rounds between 2015 and 2021 (Fig. 1). Nacala-A-Velha, Mossuril, and Ilha Mozambique were subsequently considered to have recrudescent TF as prevalence was < 5% at first TIS and then estimated to be ≥ 5% during a subsequent surveillance survey. Inhassunge was considered to have a high risk for persistent TF as prevalence had declined minimally from 19.8 to 13.6% at first TIS, in spite of implementing 2 rounds of MDA^12^.

Within each evaluation unit, a two-stage cluster random sampling design was used to sample 24 clusters (villages) with selection probability proportional to population size, using village lists provided by the Mozambique National Statistics Institute. We defined a household to be persons living together and eating from the same pot or sharing meals. Within each cluster, we selected 35 households using the compact segment method^25^. Within selected households, we interviewed heads of household and invited all residents aged 1 year and older to be examined for trachoma.

### Sample size

To estimate the evaluation unit prevalence of TF among children aged 1–9 years, we calculated the sample size assuming an expected prevalence of 4% with an absolute precision of ± 2%, 5% level of significance, a design effect of 2.63, and an inflation factor of 1.2 to account for non-response^23^. In each evaluation unit, we targeted 1164 children aged 1–9 years for examination and sampling.

### Data collection

All data were collected with standardized questionnaires on smartphones with Android operating systems using the Tropical Data (https://www.tropicaldata.org/) application and uploaded to a centralized server. Among consenting households, we collected measures on household access to water, sanitation, and hygiene facilities, GPS coordinates, and individuals’ age and gender. Conjunctival swab and dried blood spot (DBS) samples from the same individual were affixed with an identical barcode that was scanned using the smartphone camera to link the barcode with individual data.

### Clinical assessment

Graders—surveyors qualified in the WHO simplified grading system through the Tropical Data training system—conducted trachoma examination using a 2.5× magnifying binocular loupe, torch, and follicle size guides^10,11,25,26^. All consenting people aged 1 year and older were examined for TF, trachomatous inflammation—intense (TI), TT, and trachomatous scarring (TS) among those with TT. TT and TS results are outside the scope of this analysis. Trachoma grading was completed before sample collection.

### Collection of conjunctival swabs and dried blood spots

Conjunctival swab sample collection was done on the left conjunctiva by the grader and an assistant. With the left eyelid everted, the grader firmly swabbed the tarsal conjunctiva, using a sterile Dacron polyester-tipped swab, in a horizontal motion three times, rotating the swab with each motion. The swab shaft was snapped by the grader to fit the swab into a transport tube held by an assistant. Two swabs were collected per child from the same eye. One swab was stored in 1 mL DNA/RNA medium (Zymo Research, Irvine, CA, USA), and the other was stored dry. Samples were immediately placed in a cooler with ice packs for transport. The grader and the assistant cleaned their hands with alcohol gel after each examination.

A phlebotomist pricked a finger and collected blood onto filter paper (TropBio Pty Ltd., Townsville, Queensland, Australia) with 6 circular extensions calibrated to absorb 10 μL of blood. Filter papers with blood spots were placed in a covered bucket with capacity for 49 samples for transport at ambient temperature.

At the district hospital, DBS were packaged individually into sealable plastic bags and stored with desiccant in a larger bag. Swabs and DBS were transferred at the end of each day to a − 20 °C freezer and stored at − 20 °C until shipped.

### Laboratory procedures

De-identified swab samples were sent to the Proctor Foundation at University of California, San Francisco at ambient room temperature and stored at − 80 °C until processed. We used the iAMP CT Detection Kit (Atila, CA, United States). The assay is an isothermal polymerase chain reaction assay intended for the qualitative detection of Ct for endocervical swabs, vaginal swabs, and first-catch urine samples. The primer sets target the 16S plasmid regions specific to Ct. Dry samples from the same age group (1–9-year-olds) within the same cluster were randomized and pooled into groups of 10. In clusters where the number of samples was not in increment of 10s, the remaining samples were pooled into a single pool. Only pools with 5 or greater samples were tested. Briefly, 300 µL of lysis buffer was added to each dry conjunctival swab and then 50 µL of that lysis buffer was used for pooling. Five µL of the pooled sample was then used to run the iAMP Ct Detection assay as per manufacturer’s recommendations. Positive pooled samples were not tested individually. In cases where all pools from the same cluster were positive, the samples were re-pooled into 5 samples per pool and retested. This approach can accurately estimate the prevalence of Ct infection while maintaining cost-effectiveness^27^. All laboratory personnel were masked to the identity of the samples, clusters, and districts.

Filter papers with DBS were sent to the CDC at ambient temperature and subsequently stored at − 20 °C until processed. DBS were tested for antibodies against Ct antigens Pgp3 and Ct694 and a negative control antigen, Glutathione S-transferases (GST), using a multiplex bead assay (MBA) that has been previously described^14^. Each DBS extension was eluted in Buffer B (1X phosphate buffered saline (PBS) containing 0.5% casein, 0.3% Tween 20, 0.5% polyvinyl alcohol, 0.8% polyvinylpyrrolidone, 0.02% sodium azide, and 3 µg/mL *E. coli* extract) and diluted to a final serum dilution of 1:400. Beads were incubated with diluted sample for 1.5 h, washed 3 times with PBST (1X PBS, 0.05% Tween-20), and incubated with 50 ng biotinylated mouse anti-human IgG (Southern BioTech, Birmingham, AL) and 40 ng biotinylated mouse anti-human IgG4 (Southern BioTech) for 45 min. After three more washes with PBST, beads were incubated with 250 ng phycoerythrin-labelled streptavidin (Invitrogen, South San Francisco, CA) for 30 min. Beads were washed 3 more times with PBST and incubated with 0.5% BSA, 0.05% Tween-20 and 0.02% sodium azide for 30 min to remove any loosely bound antibodies. After one more wash with PBST, wells were suspended in 100 µL PBS and plates were stored overnight at 4 °C. The next day, plates were read on a MAPGIX instrument (Luminex, Austin, TX) equipped with xPONENT software (Luminex). The median fluorescence intensity (MFI) with the background from the blank well (Buffer B alone) subtracted (MFI-bg) was recorded for each antigen for each sample. Samples that had reactivity to the GST bead were excluded from the analysis. MFI-bg data were converted to dichotomous seropositive or seronegative outcomes using an MFI-bg cut-off of 295 for Pgp3 and 797 for Ct694. Values were selected to maximize sensitivity and specificity using receiver operating characteristic curve analysis on a panel of ocular Ct-PCR positive individuals from the United Republic of Tanzania (N = 122) as positives and a pediatric panel from New York, USA as negatives.

### Data analysis

We estimated district level prevalence of TF, TI, and seroprevalence of antibodies against each antigen based on mean cluster age-weighted proportions, following standard Tropical Data methods (https://github.com/itidat/tropical-data-analysis-public)^28^. Cluster mean prevalences were first drawn via random selection with replacement, and then the mean of this resulting dataset was bootstrapped with 10,000 iterations. We used the 2.5% and 97.5% centiles of the resulting distribution as 95% confidence intervals (CIs) for each prevalence measure. For each district and antibody, we fitted age-dependent seroprevalence curves using cubic splines in generalized additive models with a random intercept for cluster and estimated seroconversion rate per 100 children per year as the exponentiated intercept from a generalized linear model with binomial error structure and a complementary log–log link, using robust errors to account for survey design^29^. We examined the difference in seroconversion rates estimated using children aged 1–5 years or 1–3 years compared to 1–9 years because infections are understood to be more common in younger age groups^30^. We calculated the cluster prevalence of ocular Ct infection from pooled samples by maximum likelihood estimation such that the number of positive individual samples are most likely to have resulted in the observed pooled PCR results^27^. District prevalence of ocular Ct infection was then calculated as the unweighted mean cluster prevalence with bootstrapped 95% CIs. We classified household water, sanitation, and hygiene facilities as basic, limited, unimproved, or no facility (i.e. open defecation or surface water) according to standard definitions^31^ and estimated service level coverage from all surveyed households within the district, including those with and without children aged 1–9 years. We then plotted district service level coverage against national figures using data for 2021 obtained from the Joint Monitoring Programme (http://washdata.org). All analyses were conducted using R version 4.3.1 (https://www.r-project.org).

## Results

### Study participants

**Fig. 2:**
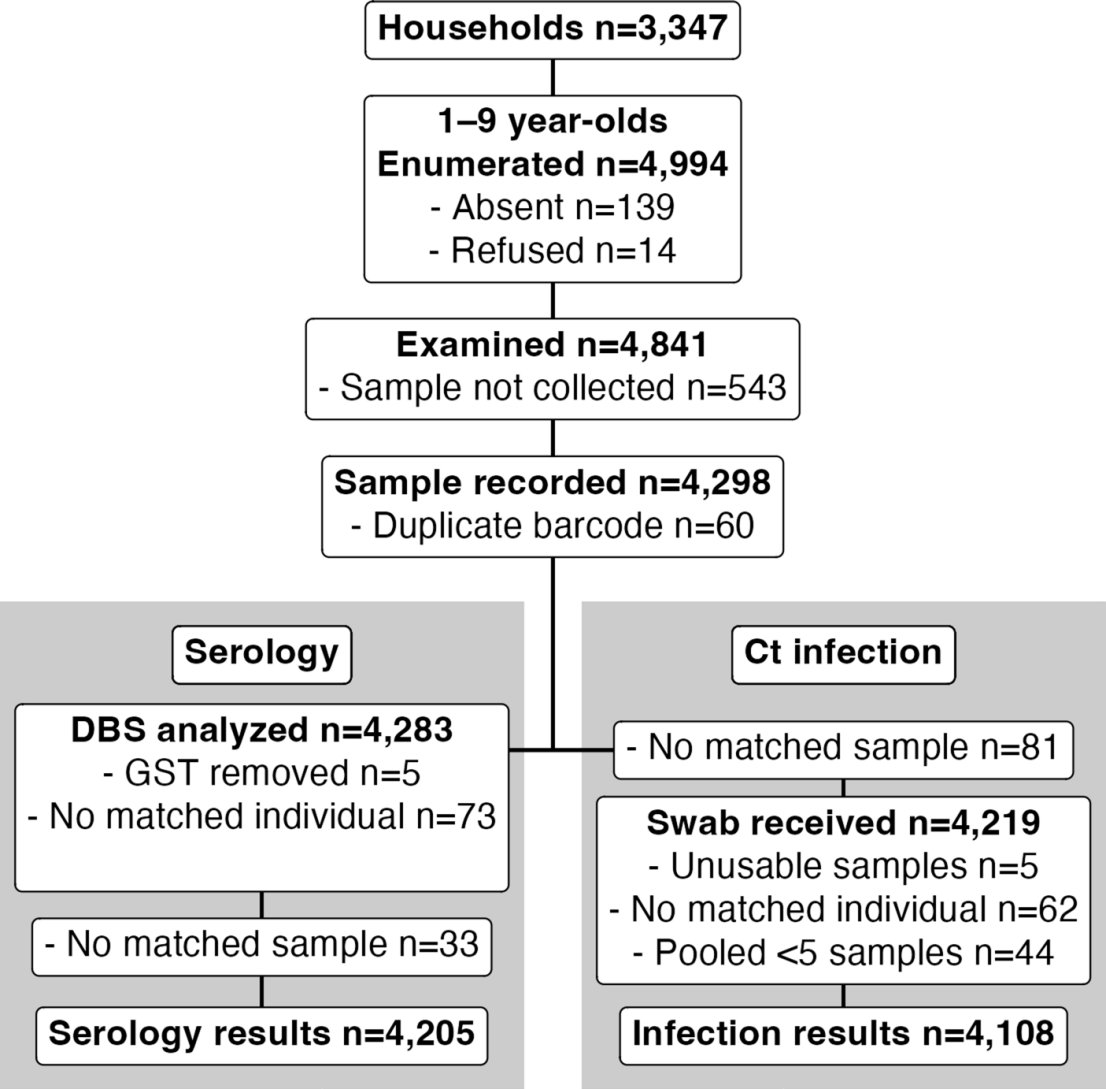
Flow charts of participants and sample collection. *DBS* dried blood spot, *Ct* ocular *Chlamydia trachomatis.*

Of 4994 children aged 1–9 years enumerated in 3347 households across 96 clusters in the four districts, we examined 4841 (96.9%) children for clinical signs of trachoma (Fig. 2). Among these children, we collected conjunctival swabs and DBS from 4298 (86.0%). We included infection and serology results from 4108 (82.2%) and 4205 (84.2%) children, respectively, in analyses.

### Indicators of trachoma in four districts of Mozambique, 2022

**Fig. 3:**
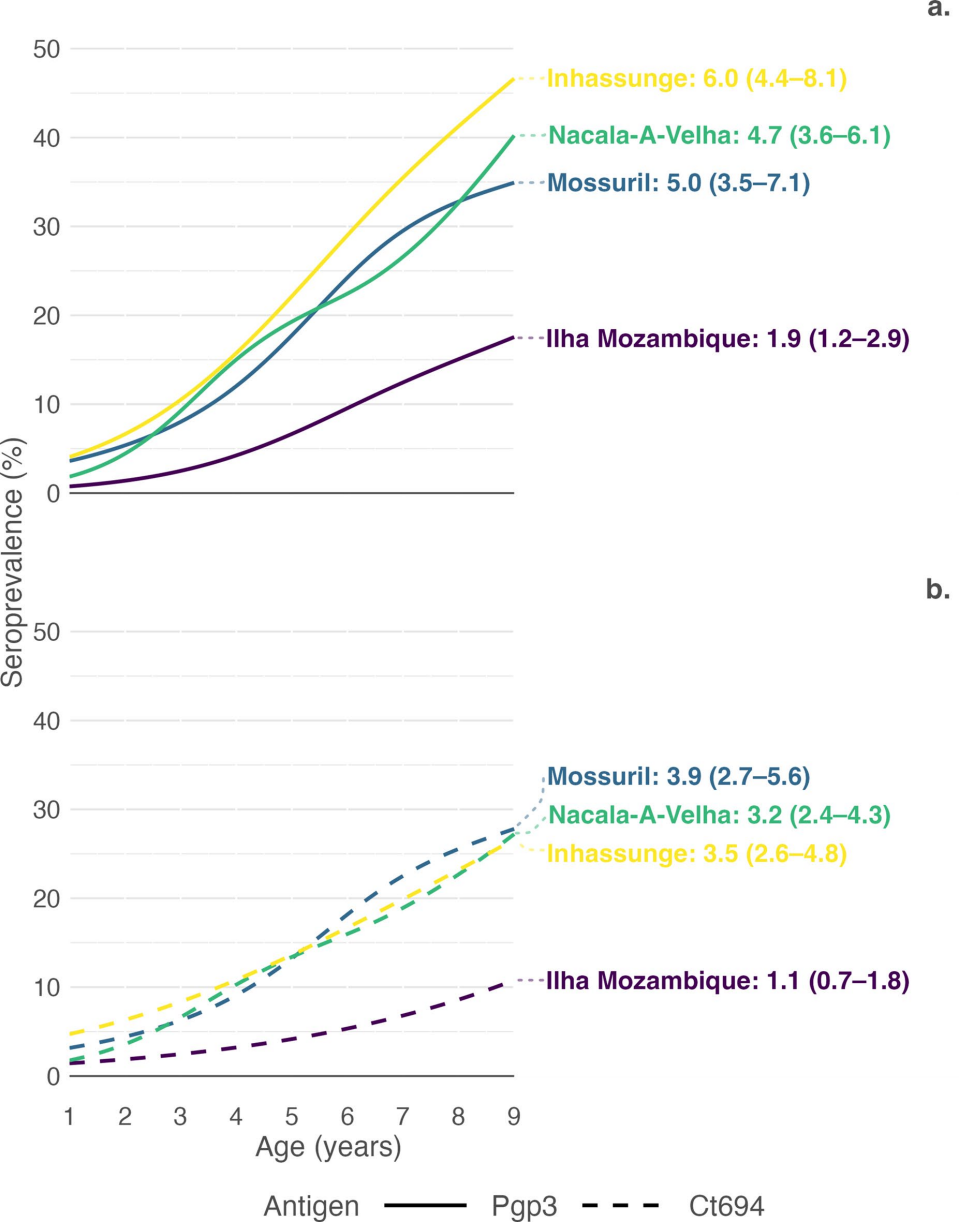
Proportion (**a**) Pgp3 and (**b**) Ct694 antibody positive by age in each participating evaluation unit. Lines are cubic splines fit to seroprevalence by age. Displayed values are estimated evaluation unit seroconversion rates per 100 person-years with 95% confidence intervals based on robust standard errors.

District measures for all trachoma indicators among children aged 1–9 years are shown in Table 1. Ilha Mozambique had the lowest observed prevalence of TF (1.1%, 95% CI 0.3–2.2) with an equivalent ocular Ct infection prevalence (1.1%, 95% CI 0.0–2.5). We found no cases of TI. We detected TF in 6/24 (25.0%) and ocular Ct infection in 4/24 (16.7%) clusters (see Supplementary Fig. S1 online). District age-weighted seroprevalence for Pgp3 antibodies was 8.8% (95% CI 5.8–11.7) and 5.5% for Ct694 antibodies (5.5%, 95% CI 3.6–7.3). Age-dependent seroprevalence curves are shown in Fig. 3 alongside estimated district seroconversion rates (repeated from Table 1). While all curves showed increased seroprevalence in older age groups, the observed seroprevalence curves for Ilha Mozambique were flatter than in other districts with seroconversion rates of 1.9 (95% CI 1.2–2.9) for Pgp3 and 1.1 (95% CI 0.7–1.8) for Ct694 antibodies.

**Table 1:** Prevalence of clinical signs, ocular *Chlamydia trachomatis* (Ct) infection, and anti-Ct antibodies and seroconversion rate among children aged 1–9 years in four districts of Mozambique, 2022.

District	Prevalence					Seroconversion rate	
	TF (95% CI)	TI (95% CI)	Ct (95% CI)	Pgp3 (95% CI)	Ct694 (95% CI)	Pgp3 (95% CI)	Ct694 (95% CI)
Ilha Mozambique	1.1 (0.3–2.2)	0.0 (0.0–0.0)	1.1 (0.0–2.5)	8.8 (5.8–11.7)	5.5 (3.6–7.3)	1.9 (1.2–2.9)	1.1 (0.7–1.8)
Mossuril	3.5 (2.1–5.3)	0.1 (0.0–0.3)	4.8 (2.2–7.4)	20.5 (15.2–26.4)	16.2 (11.6–21.2)	5.0 (3.5–7.1)	3.9 (2.7–5.6)
Nacala-A-Velha	3.6 (2.1–5.6)	0.1 (0.0–0.4)	2.9 (1.4–4.4)	18.7 (14.4–23.5)	13.0 (9.6–16.8)	4.7 (3.6–6.1)	3.2 (2.4–4.3)
Inhassunge	6.0 (2.8–8.6)	0.7 (0.1–1.4)	3.0 (0.8–5.3)	24.3 (17.8–28.4)	15.9 (11.2–18.9)	6.0 (4.4–8.1)	3.5 (2.6–4.8)

*TF* trachomatous inflammation—follicular, *TI* trachomatous inflammation—intense, *Ct* ocular *Chlamydia trachomatis* infection, *Pgp3* Antibodies against Pgp3 antigen, *Ct694* Antibodies against Ct694 antigen, *CI* Confidence Intervals based on bootstrapped sampling distribution for prevalence and based on robust standard errors for seroconversion rates.

Among the four districts, Inhassunge had the highest prevalence of TF (6.0%, 95% CI 2.8–8.6). Overall ocular Ct infection prevalence was 3.0% (95% CI 0.8–5.3), and, among 24 clusters, ocular Ct infections were detected in 11 (45.8%) clusters (see Supplementary Fig. S1 online). Pgp3 seroprevalence was highest in Inhassunge (24.3%, 95% CI 17.8–28.4) but not Ct694 (15.9%, 95% CI 11.2–18.9). Based on modeled age-dependent Pgp3 seroprevalence, 4% of 1-year-old children in Inhassunge had anti-Ct antibodies, rising to over 45% among 9-year-olds (Fig. 3). Seroconversion rate for Pgp3 antibodies was 6.0 (95% CI 4.4–8.1), and 3.5 (95% CI 2.6–4.8) for Ct694 antibodies.

We found similar prevalences of active trachoma signs in Mossuril (TF 3.5%, 95% CI 2.1–5.3; TI 0.1%, 95% CI 0.0–0.3) and Nacala-A-Velha (TF 3.6%, 95% CI 2.1–5.6; TI 0.1%, 95% CI 0.0–0.4) and observed little difference in ocular Ct infection prevalence or seroprevalence between the two districts. We detected ocular Ct infections in 13/23 (56.5%) clusters in Mossuril (swab samples were unavailable for analysis from one cluster) and 14/24 (58.3%) clusters in Nacala-A-Velha (see Supplementary Fig. S1 online). Though modeled age-specific Pgp3 seroprevalence was slightly lower at all ages than that observed in Inhassunge, seroconversion rates for both Pgp3 and Ct694 were similar between Mossuril, Nacala-A-Velha, and Inhassunge (Fig. 3).

When analysis was restricted to children aged 1–5 years and 1–3 years, estimated Pgp3 seroconversion rates were lower than in 1–9-year-olds but confidence intervals overlapped (see Supplementary Table S1 online).

### Water, sanitation, and hygiene

**Fig. 4:**
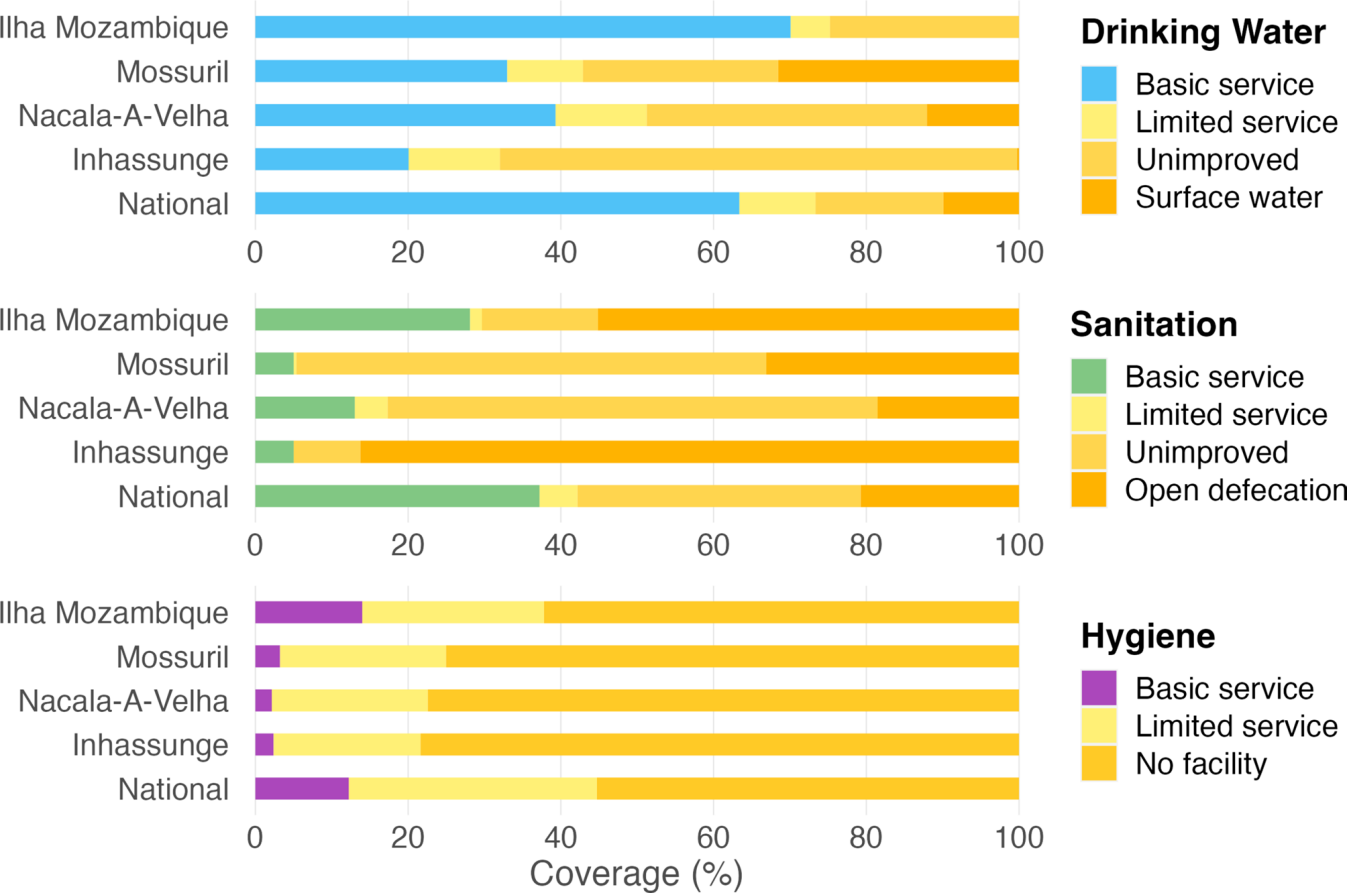
District and national water, sanitation, and hygiene service level ladders (National data for 2021 available from washdata.org). Tabulated data available in Supplementary Information (see Supplementary Table S2 online).

Reported sources of and time to collect water for drinking and washing were identical in 3267/3347 (97.6%) and 3274/3347 (97.8%) of households. Classifying household drinking water, sanitation, and hygiene facilities using the updated service level categories provides further insight into conditions within each district (Fig. 4). Reported use of an improved drinking water source within 30 min of travel (basic service) was lowest among households in Inhassunge (20.1%) in contrast to 70.1% in Ilha Mozambique and 63.4% nationally. Availability of a drinking water source within 30 min or less was highest among households in Ilha Mozambique (79.5%) and lowest in Mossuril (46.6%) (see Supplementary Table S3 online). Among 837 households surveyed in Inhassunge, 722 (86.3%) reported relying on open defecation and 817 (97.6%) had no facility or no water or soap available for handwashing (a possible marker of resources available for facial cleanliness). Hygiene facility service coverage in Mossuril and Nacala-A-Velha was comparable to Inhassunge but availability of an unimproved or better sanitation facility was higher at 66.9% (560/837) and 81.5% (682/837), respectively. Open defecation was reportedly high in Ilha Mozambique at 55.1% (461/836), which contrasts with higher than national coverage estimates for basic water and hygiene facilities.

## Discussion

We present here the results of an enhanced TIS that incorporated testing of ocular Ct infection and serology alongside an assessment of clinical signs for active trachoma in four districts of Mozambique with persistent or recrudescent TF. We found that the three recrudescent districts now had prevalence of TF below the 5% threshold for discontinuation of MDA, while the remaining district, Inhassunge, had a TF prevalence ≥ 5%, confirming it as a district with persistent TF. Triangulation, through the collection and analysis of additional trachoma biomarkers, strengthened interpretation of these TF findings^32^.

During the most recent TSS in 2019 in Nacala-A-Velha, Mossuril, and Ilha Mozambique, TF prevalence ranged between 5.2 and 5.6% (Fig. 1). TF prevalence typically lags behind prevalence of ocular Ct infection^12^, so TF prevalences > 5% in previous surveys might have been associated with little infection and may not have been responsive to further MDA. In Ilha Mozambique, we found TF prevalence well below the 5% WHO elimination threshold, reflecting the impact of further MDA of azithromycin. The observed ocular Ct infection prevalence (1.1%) was similar to those found in districts with TF < 5% in recent trachoma prevalence surveys in the Amhara Region of Ethiopia that measured ocular Ct infection among children aged 1–5 years but higher than the very low ocular Ct infection prevalence observed (0.2%) in 2014 in evaluation units in Malawi with TF < 5% among children aged 1–9 years^19,33^. Ocular Ct infection prevalence in the six evaluations units in this latter setting clearly demonstrated the lag between ocular Ct infection and TF prevalence^19^. Our estimated Pgp3 seroconversion rate (1.9 per 100 children per year) was also similar to a preliminary operational seroconversion rate threshold of 1.5 per 100 children per year found to correspond to a TF prevalence < 5% based on modeling of data from nine trachoma-endemic populations^18^. These findings support the conclusion that MDA in this district can be discontinued. Continued MDA in Inhassunge is warranted, as the observed TF prevalence of 6.0% is supported by the ocular Ct infection and serology findings. In Mossuril and Nacala-A-Velha, based on current WHO guidelines (TF prevalence < 5%), MDA should be discontinued, yet the findings from these districts are unusual. In contrast to the typically observed lag, TF prevalences in these districts remain at similar levels to prevalences of ocular Ct infection. Operational prevalence thresholds for ocular Ct infection do not currently exist, and our findings here, including seroconversion rate estimates from younger age groups, will contribute to guidance development and ongoing work to identify serological thresholds for evaluation units^18,29^. However, seroconversion rate estimates for these districts are well above the preliminary threshold, similar to the seroconversion rate observed in Inhassunge, and suggest ongoing community transmission of ocular Ct infection. Close follow-up of these populations, with an awareness that resuming antibiotic MDA may be required in the future, may be a rational approach.

Information on household water, sanitation, and hygiene (WASH) facilities is routinely collected by Tropical Data and, like the additional biomarkers, provides valuable context for understanding trachoma transmission in these settings^28^. In place of the earlier improved or unimproved facility type classification^34^, we classified reported household WASH conditions according to more recent service ladders^31^. Towards measurement of Sustainable Development Goal global targets 6.2, 6.1, and 1.4, these updated classifications highlight the proportion of the population practicing open defecation, relying on surface water, or lacking basic hygiene facilities at home. The sanitation indicator is particularly useful for evaluating the E component of SAFE^4^. Communities would benefit from trachoma control programs collaborating with relevant stakeholders to help end open defecation, as human feces is a breeding medium for the *Musca sorbens* fly, the putative mechanical vector of trachoma^35–37^. Though we have not examined the relationship at an individual level in the current analysis, lack of access to sanitation and low sanitation coverage are established factors associated with trachoma^38–40^, so the extent of open defecation in Inhassunge is a likely contributor to the persistent trachoma observed in this district. Ilha Mozambique, with the lowest prevalence of TF, ocular Ct infection, and seroprevalence, had a lower proportion of households with sanitation access than Nacala-A-Velha and Mossuril. Water availability is also related to trachoma^41,42^, but earlier classifications of improved or unimproved water sources only reflected characteristics impacting water quality, rather than quantity^34^. Service ladders now incorporate an indicator of availability based on collection time, but only among users of improved sources^31^. Alongside service ladders, the presentation of collection time, independent of water source type (see Supplementary Table S3 online) revealed differences in household water availability between districts and near exact concordance between reported drinking and washing water sources. The higher coverage of nearby water sources and likely greater availability of water for hygiene in Ilha Mozambique may balance out the impacts of open defecation, but a minimum sanitation threshold in Inhassunge does not appear to have been achieved. As per current WHO guidance, evidence suggests that F, E components of SAFE should be re-emphasized in all four district to strengthen delivery of WASH services, towards the aim of sustained elimination of active trachoma as a public health problem. Here we reported on drinking water, rather than washing water, sources and have included the standard service level ladders and information on collection time by district to facilitate conversation and coordination between the neglected tropical disease program and WASH department towards alignment of activities^43^.

Here we have demonstrated the value of incorporating testing of ocular Ct infection and serology alongside clinical grading within routine trachoma prevalence surveys. An analysis of the costs for this activity, including collection and analysis of conjunctival swabs and DBS, is forthcoming. Ct infection results were pooled, preventing analysis by age or other subgroups, and our model for seroconversion assumed homogeneity of the rate over time and age. Despite these limitations, we have confirmed that ocular Ct infection is still present and Ct transmission is likely ongoing in three of these four districts, including where TF prevalence is below the 5% elimination threshold. Analysis of household water and sanitation data furthered our understanding of likely drivers of this ongoing trachoma transmission. Subsequent prevalence surveys will confirm the importance of the levels of infection observed in these settings.

## Supplementary Information

Supplementary Information.

## Data Availability

The datasets analyzed during the current study are not publicly available but are available from the corresponding author who will submit reasonable requests for permission from the Mozambique Ministry of Health.
