# Supplementary material for: Ongoing transmission of trachoma in low prevalence districts in Mozambique: results from four cross-sectional enhanced impact surveys, 2022
Source: Sci Rep. 2024 Oct 15;14:22842. doi: 10.1038/s41598-024-71201-z (PMC11480103; doi:10.1038/s41598-024-71201-z)
Supplement: Supplementary file 1 — Supplementary Information. [file 41598_2024_71201_MOESM1_ESM.pdf]

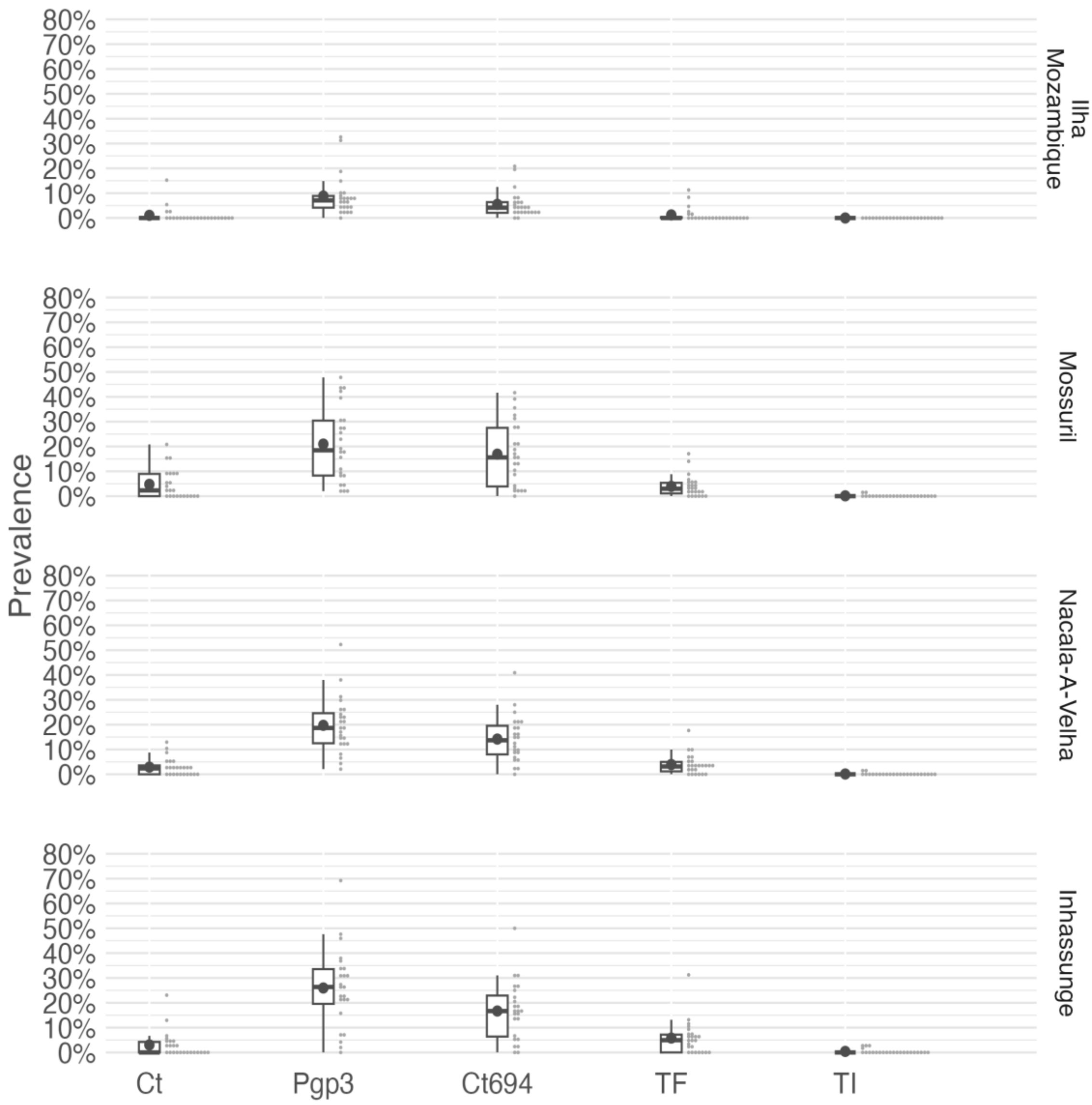

Figure S1. Distribution of cluster trachoma prevalence by district and indicator represented by box-and-whisker plots with mean cluster prevalence (dark circles) and cluster prevalence values (gray circles).

Table S1. Pgp3 seroconversion rate (SCR) by district for children aged 1–9, 1–5, and 1–3 years.

**District**	**1-9 years SCR (95% CI)**	**1-5 years SCR (95% CI)**	**1-3 years SCR (95% CI)**
**Ilha Mozambique**	1.9 (1.2–2.9)	1.4 (0.6–3.3)	1.2 (0.4–3.8)
**Mossuril**	5.0 (3.5–7.1)	4.1 (2.6–6.4)	4.1 (2.4–6.8)
**Nacala-A-Velha**	4.7 (3.6–6.1)	4.0 (2.8–5.9)	2.9 (1.7–5.1)
**Inhassunge**	6.0 (4.4–8.1)	4.9 (3.3–7.2)	5.0 (3.0–8.1)

CI = Confidence Intervals based on robust standard errors

Table S2. District and national water, sanitation, and hygiene service level coverage (National data for 2021 available from washdata.org).

Location	Households	Service Type	Basic	Limited	Unimproved	No Service
	N		n (%)	n (%)	n (%)	n (%)
**Ilha Mozambique**	836	Water	586 (70.1%)	43 (5.1%)	207 (24.8%)	0 (0.0%)
	836	Sanitation	235 (28.1%)	13 (1.6%)	127 (15.2%)	461 (55.1%)
	836	Hygiene	117 (14.0%)	199 (23.8%)	--	520 (62.2%)
**Mossuril**	837	Water	276 (33.0%)	83 (9.9%)	214 (25.6%)	264 (31.5%)
	837	Sanitation	42 (5.0%)	3 (0.4%)	515 (61.5%)	277 (33.1%)
	837	Hygiene	27 (3.2%)	182 (21.7%)	--	628 (75.0%)
**Nacala-A-Velha**	837	Water	329 (39.3%)	100 (11.9%)	307 (36.7%)	101 (12.1%)
	837	Sanitation	109 (13.0%)	36 (4.3%)	537 (64.2%)	155 (18.5%)
	837	Hygiene	18 (2.2%)	171 (20.4%)	--	648 (77.4%)
**Inhassunge**	837	Water	168 (20.1%)	100 (11.9%)	567 (67.7%)	2 (0.2%)
	837	Sanitation	42 (5.0%)	0 (0.0%)	73 (8.7%)	722 (86.3%)
	837	Hygiene	20 (2.4%)	161 (19.2%)	--	656 (78.4%)
**National**	--	Water	63.4%	10.0%	16.7%	9.9%
	--	Sanitation	37.2%	5.0%	37.1%	20.7%
	--	Hygiene	12.2%	32.5%	--	55.3%

Table S3. Frequency of collection time ≤30 minutes by water source and district, among N=3,347 households

		Districts			
**Source**	**≤30 min**	**Ilha Mozambique n (%)**	**Mossuril n (%)**	**Nacala-A-Velha n (%)**	**Inhassunge n (%)**
**Drinking water**	No	171 (20.5%)	447 (53.4%)	387 (46.2%)	229 (27.4%)
	Yes	665 (79.5%)	390 (46.6%)	450 (53.8%)	608 (72.6%)
**Washing water**	No	171 (20.5%)	466 (55.7%)	384 (45.9%)	219 (26.2%)
	Yes	665 (79.5%)	371 (44.3%)	453 (54.1%)	618 (73.8%)
